# Heating Enhancement of a Droplet on a Superhydrophobic Surface

**DOI:** 10.1038/s41598-020-61532-y

**Published:** 2020-03-12

**Authors:** Abdullah Al-Sharafi, Bekir S. Yilbas, Hussain Al-Qahtani

**Affiliations:** 1 Mechanical Engineering Department, KFUPM, Dhahran, 31261 Saudi Arabia; 2 Researcher at K.A.CARE Energy Research & Innovation Center at Dhahran, Dhahran, Saudi Arabia; 3 Senior Researcher at K.A.CARE Energy Research & Innovation Center at Dhahran, Dhahran, Saudi Arabia

**Keywords:** Engineering, Mechanical engineering

## Abstract

Enhancement of heating of a droplet on a hydrophobic surface is investigated. A vertical metal (column) pin is introduced in the droplet and the fluid heating, due to the column pin, is examined. The droplet heating is initiated at the hydrophobic surface and the column pin located in the droplet. The effect of the flow currents on the thermal fields inside the droplet fluid is assessed. An experiment is conducted to assure the velocity simulation results while using the particle image velocimetry (PIV). We demonstrated that the velocity simulations are in good agreement with the data obtained from PIV measurements. Two circulating structures are observed inside the droplet, which are related to the buoyancy and the Marangoni currents. The presence of the column pin changes the number of circulations cells to four inside the droplet. Heated fluid in region of the droplet-solid interface is transferred by the buoyancy current towards the droplet sides and heat diffusion increases temperature rise in the droplet central region. The Nusselt number attains larger values for the droplet with column pin configuration than that of the free droplet, which becomes apparent for the large droplet volumes. The Bond number improves with the presence of the column pin in the droplet; but, the Bond number values become smaller than unity for all the droplets with and without column pin configurations considered.

## Introduction

Heating of a water droplet on a hydrophobic plate results in the Marangoni and the buoyancy currents, which generate circulating structures in the droplet. The Bond and the Nusselt numbers are affected by the various parameters, some of which include droplet volume, droplet contact angle, temperature ranges, droplet fluid, and etc. The development of the Marangoni current is associated with the surface tension gradient ($\frac{d\gamma }{dT}$, where *γ* represents the surface tension and *T* being the temperature) at the droplet fluid-air interface; however, thermal expansion of the droplet fluid results in density variation, which causes the buoyancy current in the droplet fluid. The velocity ratio due to Marangoni and the buoyancy currents is similar to the ratio of the Marangoni number over the Rayleigh number^1^; therefore, the velocity ratio becomes $\frac{\frac{d\gamma }{dT}}{\rho g\beta {a}^{2}}$. For small values of the surface tension gradient $(\frac{d{\gamma }_{w}}{dT})$, the velocity ratio reduces and the Marangoni current intensity becomes small in the droplet fluid. This argument reverses for large size droplet (large values of *a*). In addition, the Marangoni convection is assessed via Marangoni number, i.e. the Marangoni influence becomes significantly important in the flow when Ma > 100^2^,. However, the relative influence of the buoyancy current over the Marangoni current can be assessed by the Bond number. In this case, Bo < 1 results the Marangoni dominance and the opposite occurs for Bo > 1. However, for the binary droplets, this argument may not be strictly applicable particularly for low evaporation temperature fluid, i.e. evaporation alters the surface tension and density variation in the droplet^3^. However, the flow behavior in the droplet can be modified through introducing the column-like structure inside the droplet, which in turn creates the conduction column while improving the droplet heat transfer characteristics. Consequently, investigation of flow behavior and heat transfer characteristics inside the droplet formed on the hydrophobic surface with the presence of vertical metallic pin is necessary.

Several investigations were realized to explore thermally disturbed droplets on the hydrophobic plates. The growth of the millimeters size droplet in a flow of steam was investigated by Yang *et al*.^4^. They showed that the shear effect on the droplet resulted in internal-flow inside the droplet. This caused heating enhancement in the fluid, which became apparent for large droplet volume. A study on the droplet entrainment in two-phase churn flow was conducted by Wang *et al*.^5^. Findings indicated that droplet formation decayed as the flow changed from churn mode to annular mode. The droplet formation was minimum within the transition of churn-annular flow regime. Heating of a droplet on surfaces with different textures were examined by Misyura *et al*.^6^. The findings revealed that the complete vaporization period for the textured surface was shorter than the plain wall. The heating rate for the textured wall remained greater than the smooth one. Thermographic investigation of the heating at the droplet-wall interfaces was conducted by Teodori *et al*.^7^. They demonstrated that the heating and the spreading was important for the droplet impact because of the reduced interaction period of the rebounding droplet. The behavior and heating of an unsymmetrical droplet in a gas flow were investigated by Legros and Piskunov^8^. They proposed a mechanism explaining the evaporation lifetimes of the droplets in a gas stream. The heating consideration for a water droplet on the superhydrophobic wetting state plate was carried out by Al-Sharafi *et al*.^9^. They showed that the heating rates enhanced with enlarging the droplet contact angle. They also introduced a new dimensionless number, Ayse, correlating the Bond number with the wetting state. Ayse number was, then, utilized developing a relation for the Nusselt number in a power law form. The droplet heating and the flow analysis were conducted by Al-Sharafi *et al*.^10^, in relation to the droplet on the hydrophobic/hydrophilic phase changing material. The findings revealed that the circulation cell center changed shifted to the solid-phase on the surface as the phase changing progressed. In addition, the Nusselt number exhibited two distinctive regions at the bottom of the droplet. Heating occurs from the liquid phase of the phase changing surface to the droplet in the first zone and, heating occurs reversely to the solid-phase on the surface in the second zone. The regional heating of the droplet for initiation of evaporation was studies by Gibbons *et al*.^11^. They indicated that the droplet evaporation occurred without altering the contact angle and heat convection was maximized around the interface of the droplet on surface. A study on the droplet heat transfer on micro-post arrays was conducted by Al-Sharafi *et al*.^12^. They showed that the pinning of the droplet on the surface was highly influenced by the micro-post arrays orientation and geometry. The Bond numbers became less than one and it demonstrates that the Marangoni effect is high in the fluid. The influence of the Marangoni current on droplet heating was studied by Phadnis and Rykaczewski^13^. They observed that conjugate heating significantly altered the fluid behavior in the droplet, which became more apparent for small droplets. The heating and fluid behavior in the droplet on hydrophilic and hydrophobic plates were investigated by Al-Sharafi *et al*.^14^. They showed that two flow cells were developed in the droplet on the hydrophilic plate. The flow cell numbers became four in the fluid as the droplet was on the hydrophobic plate. This occurred because of the joint influence of the Marangoni and the buoyancy currents in the fluid. The direction cell rotation altered as the heating direction changed. The Nusselt and the Bond numbers showed a rising trend with enhancing the contact angle. A model study for the evaporating droplet was carried out by Wu *et al*.^15^. They demonstrated that, as temperature at the wall was increased, the radius decreased and the droplet shrinkage rate became more apparent. In addition, the heating of a Leidenfrost droplet was controlled via conduction across the vapor-film. The behavior of the droplet in a rotationally flowing fluid was examined by Maneshian *et al*.^16^. They showed that the influence of flowing fluid changed the droplet behavior. Hence, the droplet experienced various shear forces, which resulted droplet deformation via stretching and shrinking. Utilization of hydrophobic nanowires towards improved droplet heating was considered by Wen *et al*.^17^. They showed that the rate of condensation influenced the droplet behavior during the nucleation phase of the droplet. Therefore, designing of such nanostructured surfaces remained important for heating enhancement.

Droplet heat transfer remains important for many thermal applications including dropwise condensation/evaporation, minimization of droplet blockage in fuel cell channels, icing/deicing of surfaces, and etc. In some applications, the hydrophobic wetting state on the surface becomes necessary due to heating enhancement such as dropwise condensation/evaporation. Although heating of a droplet on a hydrophobic wetting state plate was studied previously^9,10,12,14^, the main focus was the assessment of heat transfer characteristics of a sessile droplet located on the hydrophobic surfaces. The droplet heat transfer improvement via insertion of a high thermal conductive pin or placement of a column-like structure inside the sessile droplet was left for forthcoming investigation. The presence of the column-like thin metallic wire changes the droplet pinning characteristics on the surface and influences the heating rates of the fluid. The heat transfer enhancement without altering hydrophobic characteristics of the surface becomes critical for the droplet thermocapillary behavior; hence, the Marangoni and the buoyancy current intensities can vary in the droplet fluid. The wetting state of the pin surface remains critical for the heat transfer enhancement of droplet heating. The hydrophobic pin results in interfacial resistance between the droplet fluid and the pin surface because of the surface texture. In order to avoid the thermal boundary resistance, due to texture on the pin surface, between the droplet fluid and the pin surface, the wetting state of the pin surface needs to be kept hydrophilic. Consequently, in the present study, internal fluidity and heat transfer of a sessile droplet on the hydrophobic surface with the presence of column-like thermally conductive hydrophilic pin are examined. The thermal fields are predicted for different droplet volumes in accordance with the experiment. The velocity simulations are compared with those of the particle image velocimetry experiments. The Bond and the Nusselt numbers are evaluated for different droplet volumes and the findings are compared with those obtained for the sessile droplets on the hydrophobic surface without presence of the column-like structure.

## Mathematical Analysis and Numerical Solution

Heat and flow analysis of a water droplet on the hydrophobic surfaces is considered and the effect of vertically placed pin inside water droplet is examined. The data obtained from the initial simulations for occurrence of the quasi-steady thermal behavior in the liquid is in the order of 30 s. Although 30 s is a small time period, the droplet evaporation triggers the vapor-phase formation at the liquid-air-interface. The vapor transfers towards the air ambient can be written via the diffusion-convection equation,

1 $$\frac{\partial {C}_{v}}{\partial t}+\nabla \cdot (-D\nabla {C}_{v})+{u}_{g}\cdot \nabla {C}_{v}=0$$

here, *u*_*g*_ represents the velocity of the gas phase, *D* is the diffusion coefficient (m^2^s^−1^) and *C*_*v*_ is the specific heat capacity under constant volume (kJ/kgK). For a sessile droplet drying study, the droplet weight-loss is considered to evaluate the amount of evaporation from the spherical droplet free surface. For the evaporating surface, tracking the droplet fluid-air-interface remains important. In the numerical treatment, an approach of moving mesh incorporating the Arbitrary Lagrangian-Eulerian (ALE) technique becomes fruitful as compared to that utilized volume of fluid technique^18^. Heating and flow are coupled and incompressible flow situation is considered. The continuity equation is:

2 $$\nabla .V=0$$

here *V* represents the velocity.

The momentum equation can be expressed as:

3 $$\rho (\frac{\partial V}{\partial t}+V\cdot \nabla V)={\rho }_{o}\beta (T-{T}_{o})g-\nabla (p-{p}_{o})+\nabla \,[\mu (\nabla \,\mu +{(\nabla \mu )}^{{\rm{t}}})]$$

here *p* represents the pressure, *μ* is the viscosity of the liquid, *g* corresponds to acceleration due to gravity and *p*_*o*_ corresponds to the hydrostatic pressure relating to density *ρ*_*o*_ and temperature *T*_*o*_ and t is the matrix transpose operator. The variation in density takes place due to liquid thermal expansion and it could be expressed using the Boussinesq approximation, i.e.:

4 $$\rho ={\rho }_{o}(1-\beta (T-{T}_{o}))$$

here *β* is the water thermal expansion.

The energy equation yields:

5 $$\rho {C}_{p}\frac{\partial T}{\partial t}+\rho {C}_{p}V.\nabla T=\nabla .(k\nabla T)$$

here *C*_*p*_ corresponds to the specific heat and *k* represents the thermal conductivity.

The Bond number (*Bo*) is the force ratio due to buoyancy over the surface tension, i.e: $Bo=\frac{\beta g\rho {a}^{2}}{|d\gamma /dT|}$, here *γ* is the surface tension while *a* corresponds to the characteristic-diameter (length scale), which is $a=\frac{{V}_{d}}{\pi {R}_{w}^{2}}$, here, *V*_*d*_ represents the volume of the droplet and *R*_*w*_ corresponds to the wetting radius.

The Grasshoff number (*Gr*) is related to the natural convection and it is $Gr=\frac{\beta g\varDelta T{a}^{3}}{{\upsilon }^{2}}$, here ΔT represents the temperature and ν is the kinematic viscosity. The Marangoni number (*Ma*) is $Ma=\frac{|d\gamma /dT|\varDelta Ta}{\mu {\alpha }_{T}}$, here *α*_*T*_ represents the thermal diffusivity. It should be noted that when *Bo* < 1, the internal flow is dominated mainly by the Marangoni convection; however, the natural-convection effect on the flow becomes negligible for *Gr* < 2400^2^.

## Initial Conditions

The droplet is initially assumed at 300 K, and the hydrophobic surfaces as well as the vertically located (column) pin are kept at 304 K. The fluid velocity is considered to be zero initially resembling the droplet fluid stagnation. The air at atmospheric pressure (101.32 kPa) and temperature (300 K) is assumed for droplet exterior.

## Boundary conditions

**Figure 1:**
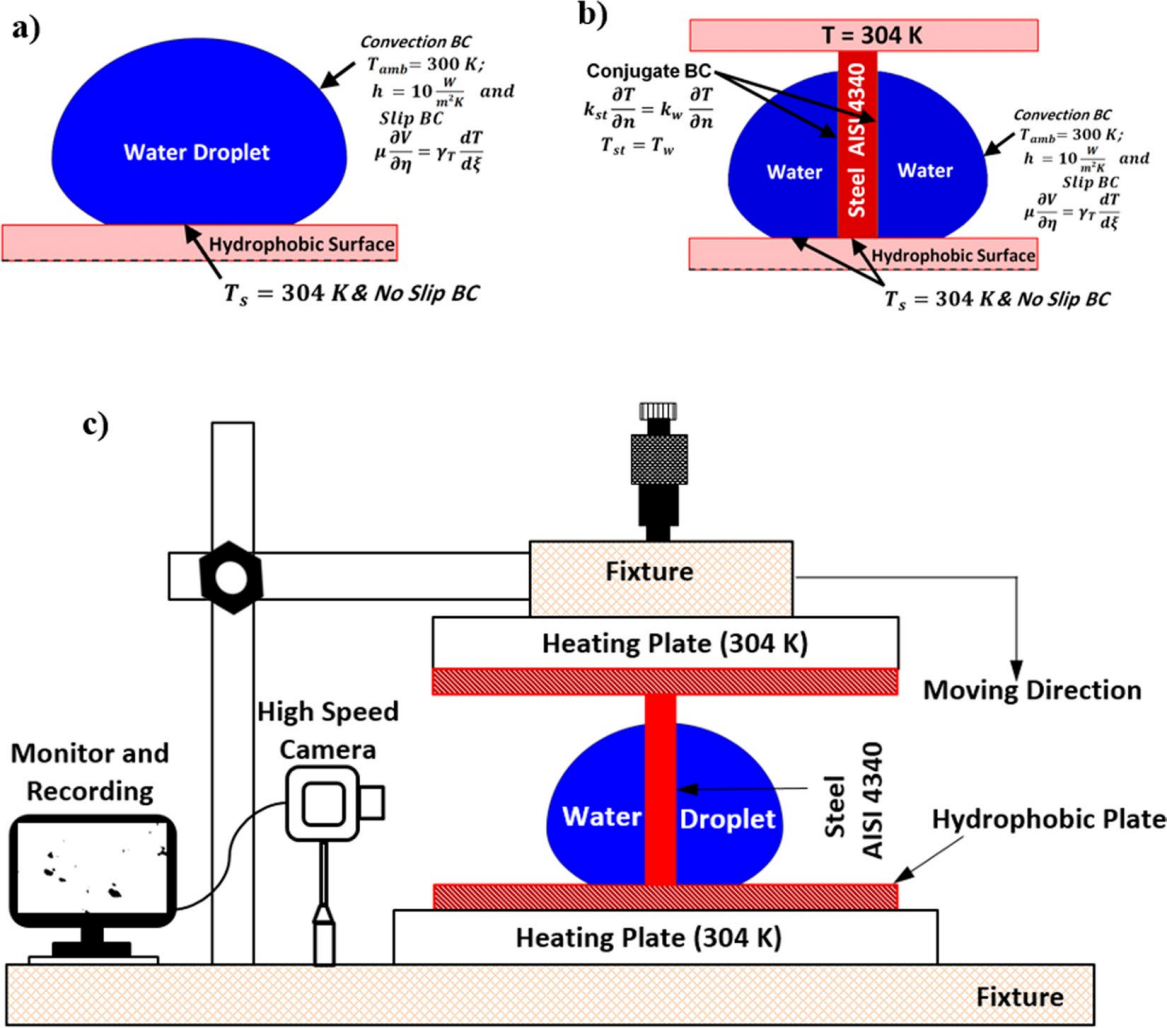
A schematic view of a droplet: (**a**) boundary conditions used in the simulations for case without column pin, and (**b**) boundary conditions used in the simulations for case with column pin presence, and (**c**) experimental set up.

Figure 1a,b show the boundary-settings used in the computation for the free droplet on the hydrophobic plate (Fig. 1a) and the vertically located column pin in the droplet (Fig. 1b) while Fig. 1c shows schematic view of the experimental setup. The boundary conditions were set according to the experimental conditions. The droplet fluid-air interface is set at atmospheric conditions. The natural-convection and radiation boundary was used at the water-air-surface. The un-wetted plate surface (surface not covered by the droplet), the natural-convection and radiation conditions were adopted. The hydrophobic-plate was set at 304 K and the conjugate heating was introduced over pin-droplet interface. A slip boundary was incorporated at the interface of droplet-air, i.e. the shear stresses were considered to be in a same order at the interface, i.e. the slip boundary, which adopted the none-viscous influence on the slip surface (no-boundary-layer is formed). Moreover, the mass balance across the interface can provide the rate of evaporation. In line with the early work^19,20^, it yields:

6 $$\{\begin{array}{c}J={\rho }_{w}({u}_{w}\cdot n-v)\\ J={\rho }_{g}({u}_{g}\cdot n-v)\end{array}$$

here *v* represents the vapor front velocity at the air-liquid interface, which is normal to the free surface, and n is the normal unit vector (normal to the surface of interface), which points outward from the interfacial surface. The subscripts *w* and *g* represent the droplet fluid (water) and ambient air (gas), respectively. The velocity jump (*u*_*w*_ − *u*_*g*_) at the interface due to liquid and gas phases can be expressed as with the support of mass-flux across the interface, i.e.:

7 $$({u}_{w}-{u}_{g})\cdot n=J\frac{1}{{\rho }_{w}}-\frac{1}{{\rho }_{g}}$$

The droplet shrinking velocity becomes:

8 $$v={{\boldsymbol{u}}}_{w}\cdot n-\frac{J}{{\rho }_{w}}$$

The shrinking velocity remains normal to the gas-liquid interface and the tangential component of the shrinking velocity is zero. Moreover, during the surface evaporation, the velocity discontinuity occurs at the interface due to differences in density of liquid and the surrounding environment. Hence, evaporation gives rise to momentum flux in terms of the tension forces at the interface, i.e surface stresses at the interfac^21^. The analogy of Marangoni shear can be adopted to account for the interfacial stress, which serves as the boundary condition for the momentum equation at the interface (droplet free surface). Hence, the consideration of the stress balance at the interface (liquid-air) yields:

9 $${(n\cdot {\boldsymbol{{\rm T}}})}_{g}={(n\cdot {\boldsymbol{{\rm T}}})}_{w}+S{T}_{f}$$

where **T** is the total stress tensor (N m^−2^), which is ${\boldsymbol{{\rm T}}}=-\,pI+\mu (\nabla u+{(\nabla u)}^{{\rm{t}}})$, superscript t is the matrix transpose operator and *ST*_*f*_ is the surface tension force, which is:

10 $$S{T}_{f}=\gamma ({\nabla }_{\varGamma }\cdot n)n-{\nabla }_{\varGamma }\gamma $$

here $\gamma ({\nabla }_{\varGamma }\cdot n)n$ is the force per unit area due to droplet curvature at the interface $({\nabla }_{\Gamma }\cdot n)$, and ${\nabla }_{\varGamma }\gamma$ is the tangential stress compound related to the surface tension gradient. The tangential stress compound at the interface can be written as:^19,20^

11 $${\nabla }_{\Gamma }\gamma ={\gamma {\prime} }_{T}{\nabla }_{\Gamma }T$$

where ${\gamma {\prime} }_{T}$ is the surface tension gradient with respect to temperature (N m^−1^K^−1^), and ${\nabla }_{\varGamma }T$ is the temperature gradient on the surface at the interface. The local mass flux equation is coupled with the momentum equation estimating the vapor flux at the interface. The mass balance at the interface yields the vapor mass flux, which is normal to the interface, must be same as the mass flux of liquid normal to the interface, i.e. the evaporated liquid is same as the vapor content at the interface. Moreover, the transfer of vapor phase to the droplet ambient (air environment) across the interface can take place by convection and diffusion. Hence, the convective and diffusive fluxes can be written as^20^:

12 $$J={\rho }_{w}({u}_{w}\cdot n-\upsilon )={\rho }_{v}({u}_{g}\cdot n-\upsilon )-{D}_{v-a}\nabla {\rho }_{v}\cdot n+{\rho }_{a}({u}_{g}\cdot n-\upsilon )-{D}_{a-v}\nabla {\rho }_{a}\cdot n$$

The vapor and ambient air mass fluxes are included on the right hand side of Eq. (12)^20^. The equation for the vapor mass flow across the interface yields^21^:

13 $$J={\rho }_{v}({u}_{g}\cdot n-\upsilon )-D\nabla {\rho }_{v}\cdot n$$

here *D* is the diffusion coefficient and *D*_*v* – *a*_ = *D*, the subscripts *v*−*a* and *a*−*v* represent vapor phase in ambient air and air (gas) in the vapor phase, respectively. The mass loss of the droplet during 100 s is found to be about 0.12%. To verify the amount of weight loss of the droplet, the droplet geometric features are measured for the heating period of 100 s. In this case, the high speed imaging (Dantec Dynamics SpeedSense 9040) is used recording the droplet geometric image every-after 0.1 s. Through this arrangement, it is possible to estimate the volume change of the droplet via data recorded from the high speed camera for the period of 100 s. The changes of the geometric feature of the droplet are determined over the heating period (100 s). The results show that the droplet volume change becomes smaller than 0.1% during heating period of 100 s. This can apply for all the volume of the droplets used in the study (30 µL–90 µL). Moreover, the droplet evaporation is determined and the fluid evaporated, which is calculated as 0.07652 × 10^−3^ grams at the end of 100 s of heating for 50 μL droplet at the atmospheric ambient condition (300 K and 85% of the air relative humidity, which is same as the experiment). The mass loss of the droplet fluid (water) because of evaporation is in the order of 0.15% of the total mass of the water droplet. Consequently, no significant droplet mass loss occurs due to the evaporation from the droplet surface for the short heating duration considered in the present analysis.

**Figure 2:**
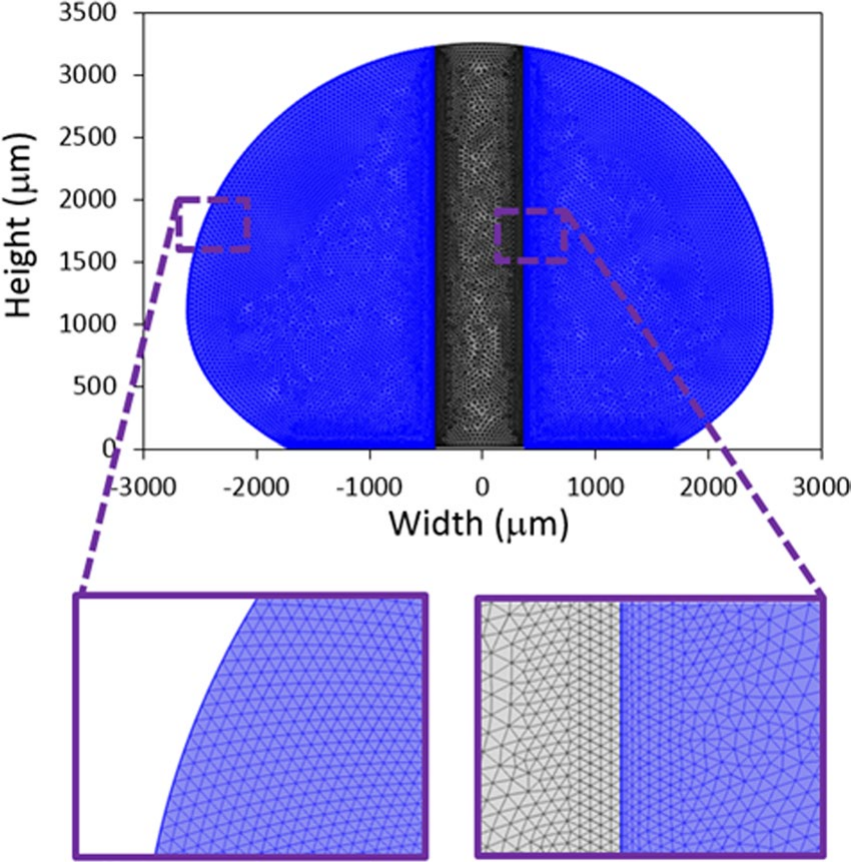
Mesh distributions.

**Figure 3:**
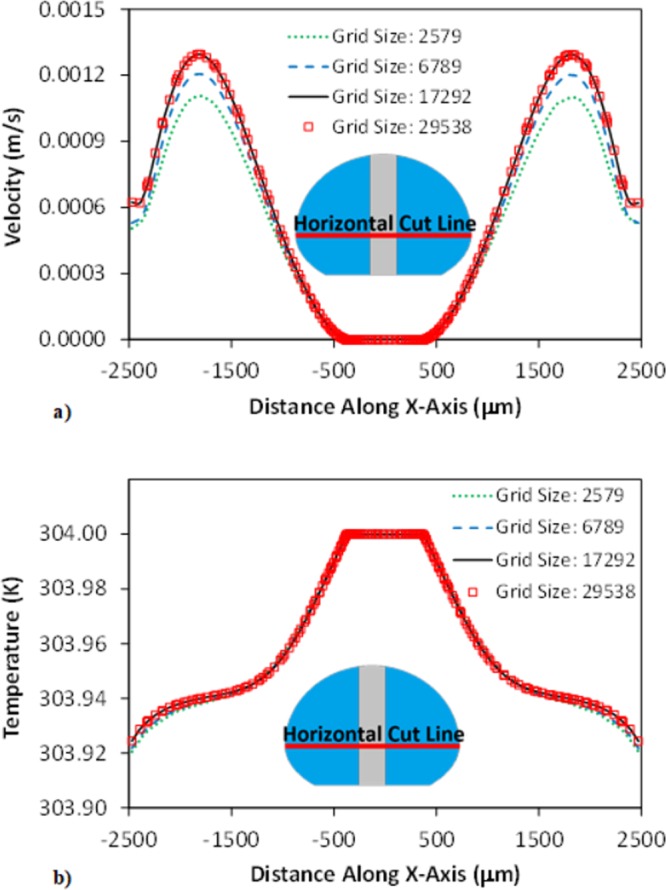
Grid independent test findings: (**a**) velocity variation in horizontal line for various meshes, and (**b**) temperature variation in horizontal line for different meshes.

A numerical code (COMSOL Multiphysics^22^,) was used to simulate the flow and temperature fields inside the droplet fluid located on the hydrophobic surfaces. In line with the experimental conditions, the droplet volumes were varied within 30 μL to 90 μL in the simulations. The variation of the droplet geometries was recorded by the high-speed camera (Dantec Dynamics (SpeedSense 9040)) during the heating period. The data for the droplet geometry corresponding to the free droplet and droplet with the vertically located column pin on the hydrophobic surfaces were evaluated. The recorded droplet geometries were re-established mimicking the imaged droplet geometry for the cases of with and without the column pin. However, 3-D computation of the thermal field in the droplet was costly due to large grid-points requirements for the correct results; hence, 2-D axisymmetric computations was realized predicting droplet heating. The small size grids were located in the places with high fluxes. The grid independence tests were realized securing the grid independent solutions. Figure 2 depicts the grids utilized in the computations and Fig. 3 depicts the results of the grid size tests for velocity (Fig. 3a) and temperature (Fig. 3b) variations over the horizontal-cut-line for 50 μL droplet. After completing the grid independent tests, the grid size of 17292 points was chosen and used in the computations. The backward Euler finite difference technique was utilized discretizing the flow equations. The implicit method was utilized and unconditionally stable solutions were obtained^23^. The time step for the computations was crucial ensuring the computation accuracy; hence, it was fixed at 10^−8^ s. The residuals of all parameters are fixed as $|{\psi }^{k}-{\psi }^{k-1}|\le {10}^{-8}$.

## Experimental and Characterization Tools

A hydrophobic plate surface is created by using the functionalized silica-nanoparticles, which were deposited onto a glass plate. The nanoparticles were synthesized/functionalized adopting the procedure of the early study^24^. Tetraethyl orthosilicate (TEOS), 3-aminopropyltrimethoxysilane (AMPTS), isobuthytrimethoxysilane (OTES), ethanol, and ammonium hydroxide were used. The mixture solution was mechanically shaken over 15 hours in laboratory conditions. Later, the mixture was treated, via centrifuging and washing, while satisfying all residual reactants removal. The dip coating technique was adopted for the deposition of functionalized-nanoparticles on to the glass surface. The solvent residues on the coated surface was vaporized via vacuuming. The functionalized silica-nanoparticles deposited surfaces were, then, characterized using the analytical tools including scanning electron microscope (SEM) and atomic force microscope (AFM) line scan.

**Figure 4:**
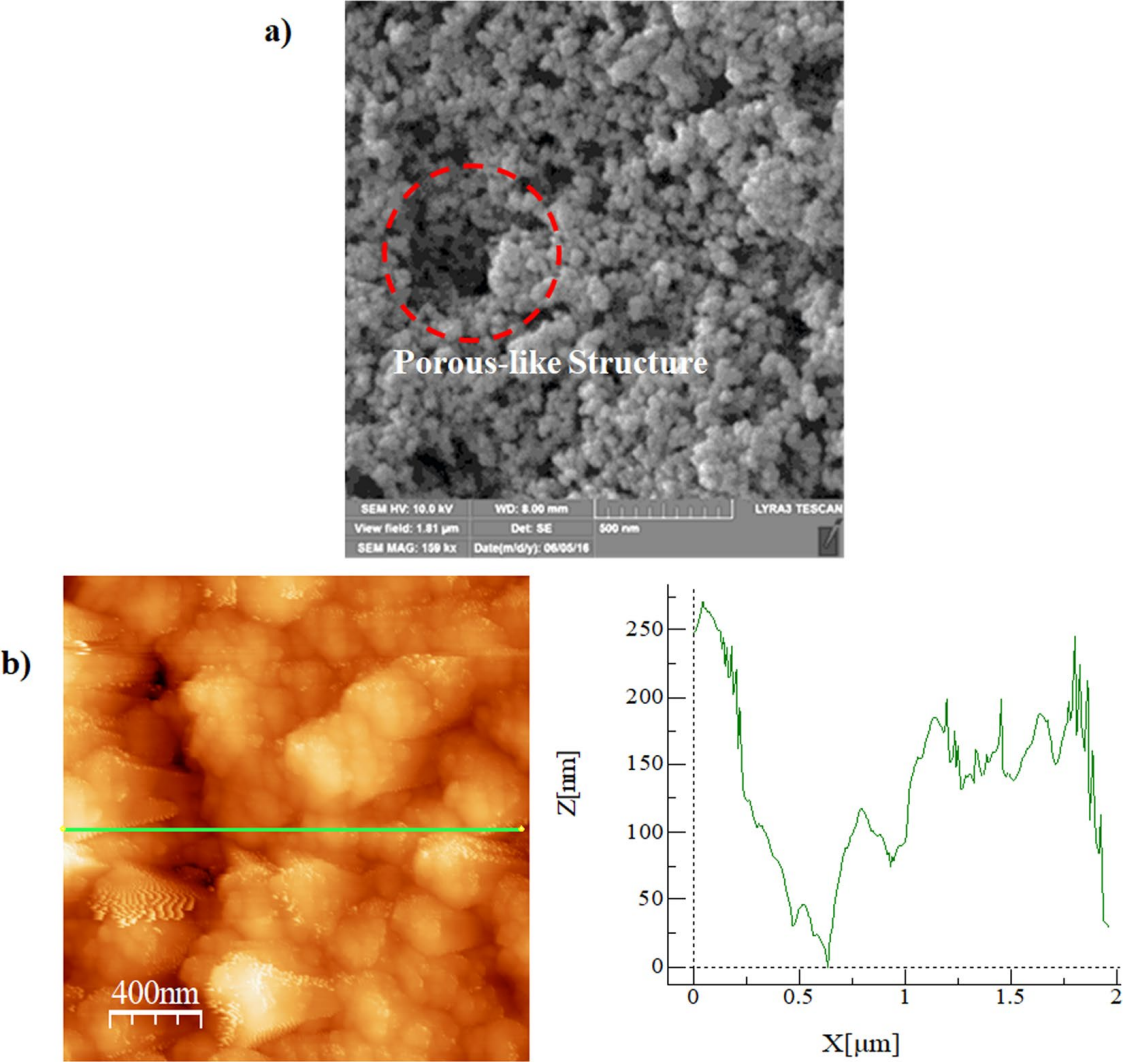
SEM micrograph and AFM line scan of functionalized silica nanoparticles coated surface: (**a**) SEM micrograph. The dotted circle shows the porous-like texture, and (**b**) line scan along the surface.

Characterizations of the resulting samples were realized utilizing scanning electron microscope (SEM, JEOL 6460), atomic force microscope (AFM), optical microscope, and goniometer (Kyowa, model - DM 501). A care was taken obtaining the high resolution and stable images for the contact angle measurements^25^. Coated samples were gold plated prior to obtain SEM micrographs of surfaces. In the case of AFM surface texture analysis, coated samples were cleaned ultrasonically to eliminate the loose silica particles. Figure 4a depicts SEM micrograph of functionalized silica nanoparticles deposited glass plate and Fig. 4b depicts the line scan of texture data resulted from atomic force microscopy (AFM) for same surface. The use of tetraethylorthosilicate (TEOS) towards synthesizing the silica-particles results in agglomeration of the synthesized silica nanoparticles on the surface. This is because of the growing condensing monomer, i.e., they form at a faster rate than that of the nucleation. This, in turn, lowers the new nuclei formation rate and agglomerate increase of the synthesized-particles^24^. In addition, the hydroxyl groups may have different moieties on silica surfaces while altering the side reactions, i.e. causing the condensation hydroxyl groups on the surface of the nanoparticles^26^. The deposited nanoparticles can result in clusters on the glass plate. This results in enhancement of the collection of the nanoparticles while forming the porous-like-structures on the deposited surface (Fig. 4a). However, the size of porous-like structures are small and do not extend to entire surface. The area coverage of these structures is in the order of 5% over the entire surface. The porous-like structures modify the surface texture and forms small size cavity at the surface, which is observed from Fig. 4a. These texture sites provide gaps at the surface, which are enable to trap air in between the droplet and the surface. The functionalized silica particles forms nano-size pillars on the deposited surface (Fig. 4b). In addition, the wavy appearance of the line scan of the surface is associated with the closely spaced functionalized silica particles in the agglomerated region of the surface. The average surface roughness is about 155 nm and the roughness parameter is in the order of 0.32. The parameter for roughness can be described by the area ratio of pillars over the area of the projection area^27^.

A mechanical-fixture was designed and realized setting the column pin vertically; in which case, a micrometer was used in the fixture to adjust the vertical position of the pin. The hydrophobic glass surface and column pin were kept at 304 K for two hours via a constant temperature plate. The ambient air conditions of the experiment are monitored (temperature and the relative air humidity). The ambient air condition was about 300 ± 0.1 K with relative humidity within 85% ± 0.1%. A high speed optical recording unit (Dantec Dynamics SpeedSense 9040) was utilized monitoring the droplet geometric feature.

## Validation of Velocity Predictions through Velocity Comparison

**Figure 5:**
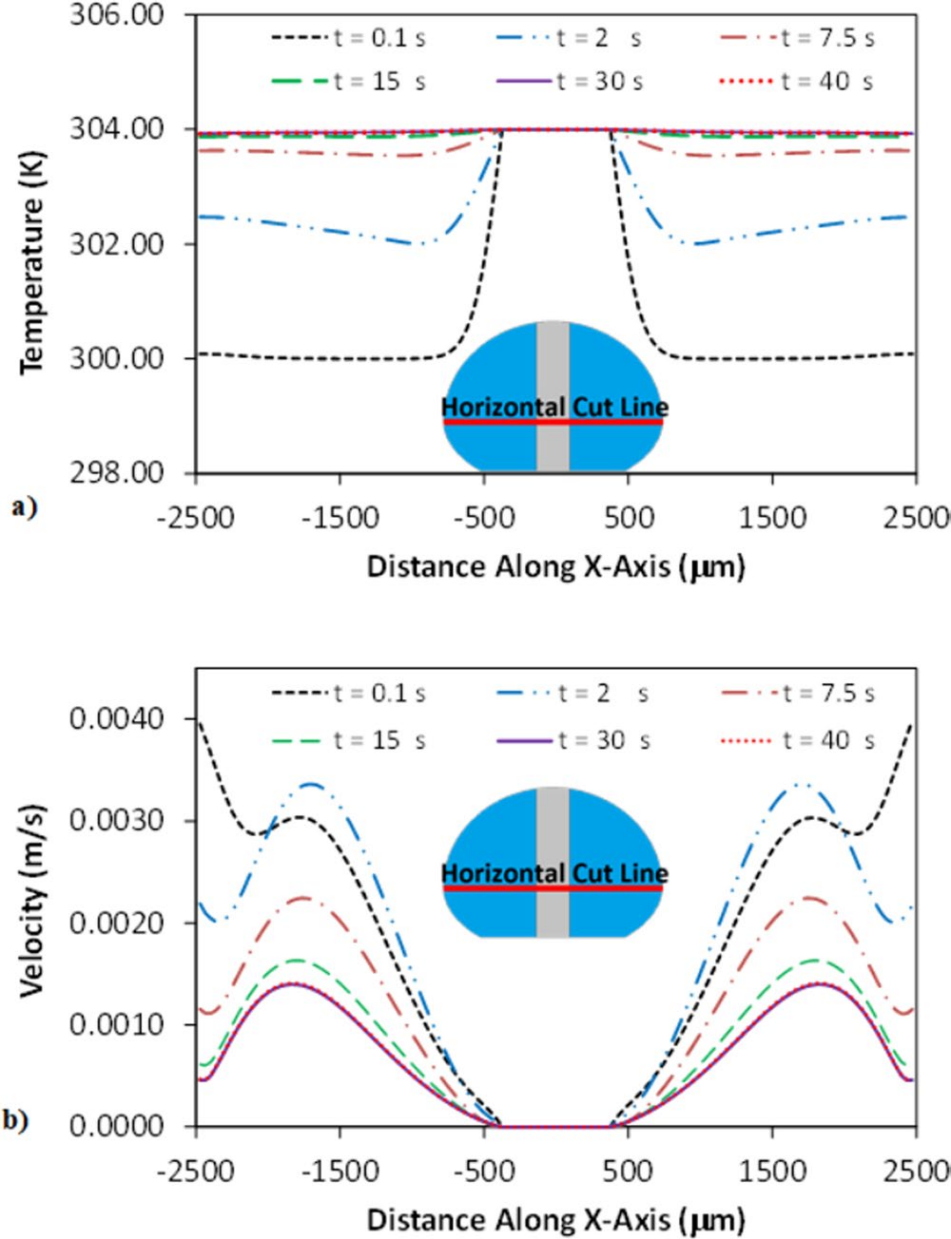
Temperature and velocity variation along the horizontal line for various heating durations: (**a**) temperature variation, and (**b**) velocity variation.

**Figure 6:**
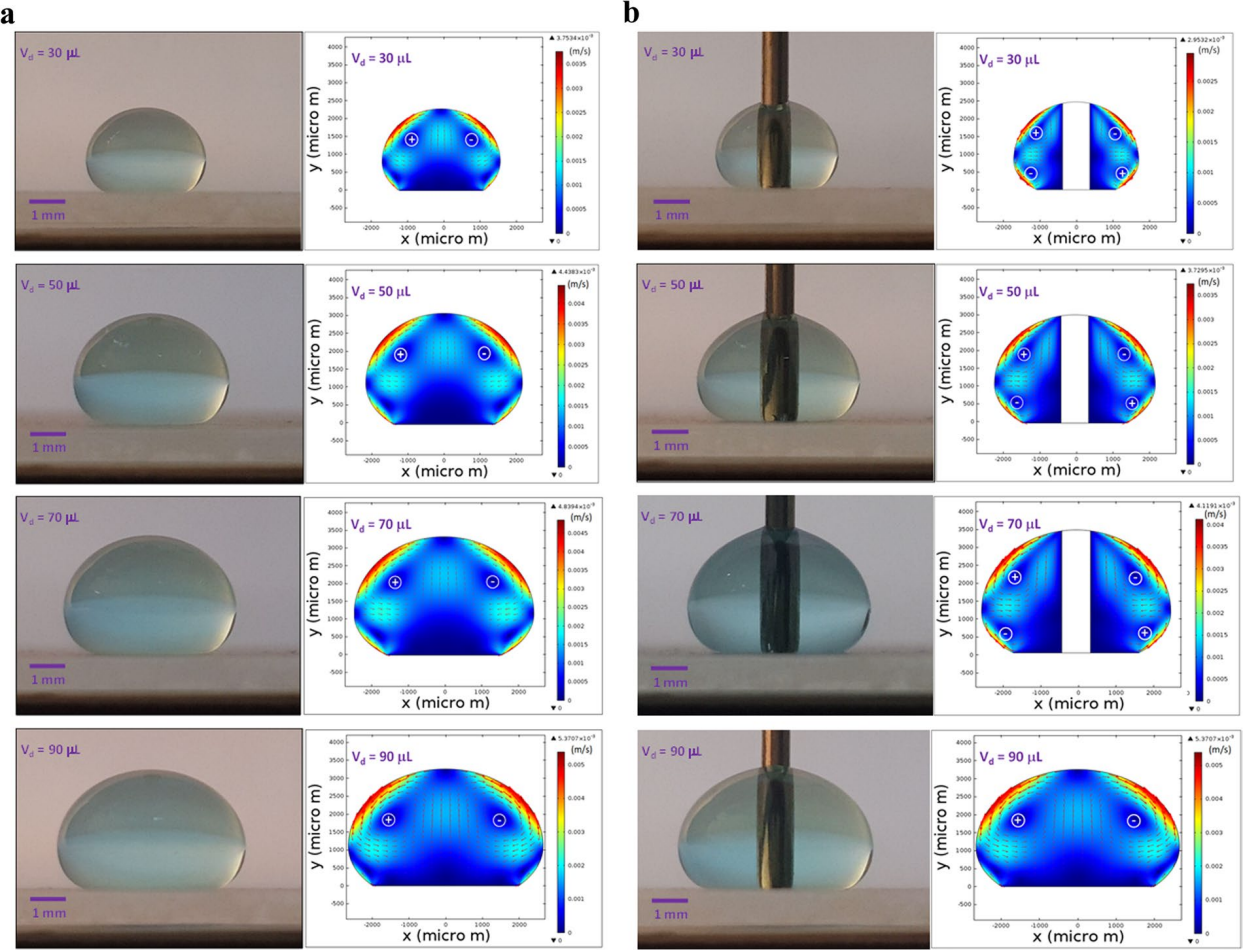
(**a**) Optical image of the droplet and velocity contours inside droplet without column pin (sessile droplet) for different volumes after 40 s of heating. Circles in the velocity contours represents circulation cells formed inside droplet and +ve corresponds to counterclockwise while −ve corresponds to clockwise rotations. (**b**) Optical image of the droplet and velocity contours inside droplet with presence of column pin for various volumes after 40 s of heating. Circles in the velocity contours represents circulation cells formed inside droplet and +ve corresponds to counterclockwise while −ve corresponds to clockwise rotations.

The flow velocity predicted was validated via particle image velocimetry (PIV) data. Carbon nanotubes (CNTs) and water mixture (0.1% CNT by volume) was used to form a droplet. Moreover, PIV experiments are repeated incorporating 1% hollow glass particles with 5 µm sizes. However, CNT formed cluster-like structures in the droplet fluid, which allowed easiness to record the locations of the particles in the droplet fluid using the high speed camera. The average size of the cluster-like CNT structures was in the order of 1 µm; however, the size of the few structures was larger than the average size, i.e. they are about ∼5 µm. These appeared to be only few large structures in the fluid and they made it easy to follow flow field via high speed recording. Several tests were carried out without CNT clusters while incorporating the hollow glass particles (0.1% concentration by volume) in the droplet fluid. The findings revealed that the clustered CNT did not influence the flow field. This could be because of low concentration (0.1% CNT by volume) and low density of CNT in the droplet fluid. Moreover, the pin made from steel had 0.75 mm in diameter, which was placed on the hydrophobic surface via penetrating 70 μL water droplet by using a fixture. The pin and plate temperatures are kept at 304 K. The particle image velocimetry (PIV) was utilized evaluating the motion of CNT-clusters in the droplet. The flow and temperature fields are computed using the mixture properties via resembling the experiments. The flow and energy equations were solved numerically adopting the slurry single phase fluid. The formulations of properties of the mixture and flow equations are refereed to early work^28^. The motion of the CNT clusters was monitored by high speed imaging recording with the recording rate of 100 fps and 960 × 720 pixels resolution. The optical distortion due to droplet curvature was corrected utilizing the correction method provided in the early work^29^. The tracker program was used to track the individual clustered-particles with time and velocity of the induvial clusters was obtained from the tracker program at different locations inside the droplet fluid. The measurement tests were repeated ten-times securing the measurement repeatability. The uncertainty involvement with the cluster-displacement, in pixels, was estimated to be 0.05, which was similar to that presented in the early study^30^. Temperature and velocity variations in the droplet are simulated at various times as shown in Fig. 5a,b. Temperature becomes almost same along the horizontal axis (Fig. 6a) after 40 s of heating time. Hence, the measurements are carried out after 40 s. However, the measurements are repeated for 60 s and 100 s of heating times to ensure the repeatability of the measurement data. The error related to the measurement repeats is found to be about 5%; hence, the data obtained from the measurements remain almost same after 40 s of heating duration. Table 1 gives the velocities obtained from PIV and predicted from simulations. The velocity of the particles resulted from the experiment and simulated are in a good agreement. Some minor differences of findings are because of the simulation errors, i.e. round-off errors, and that of the errors resulted from the experiment, which is estimated as 5%.

**Table 1:** Simulation and measurement data for particle velocity at different locations in the fluid.

Particle #	X (μm)	Y (μm)	Simulation *V*_*S*_ (m/s)	Experiment *V*_*E*_ (m/s)	|Difference| |V_*s*_−*V*_*E*_| (m/s)	% Difference		
1	663.364	1757.658	0.000728	0.000723	5.1 × 10^−6^	0.636	Simulation	
2	602.505	2487.967	0.000980	0.000982	2.3 × 10^−6^	0.174		
3	906.800	2962.668	0.000826	0.000830	4.2 × 10^–6^	0.500		
4	1357.157	2804.434	0.001825	0.001822	3.2 × 10^−6^	0.151		
5	1868.374	2195.844	0.001608	0.001600	8.1 × 10^−6^	1.941	CNT Particle Tracing Inside Water Droplet	
6	1953.576	1721.143	0.001056	0.001052	4.3 × 10^−6^	0.407		
7	1600.593	1404.676	0.001365	0.001369	4 × 10^−6^	0.261		
8	1113.721	1173.411	0.000690	0.000682	8.2 × 10^−6^	1.232		
9	894.628	406.587	0.000236	0.000241	5.3 × 10^−6^	1.915	Particle Initial Location	
10	1564.078	236.181	0.001110	0.001103	7.1 × 10^−6^	0.548		
11	2123.981	516.133	0.001994	0.001998	4.2 × 10^−6^	0.180		
12	−565.989	1940.235	0.000659	0.000666	7.2 × 10^−6^	1.000		
13	−614.677	2548.826	0.001019	0.001016	3.3 × 10^−6^	0.341	Particle Intermidate Location	
14	−553.817	3157.417	0.000631	0.000625	6.2 × 10^−6^	0.982		
15	−1101.549	3047.870	0.001889	0.001884	5.1 × 10^−6^	0.248		
16	−1783.171	2573.170	0.003122	0.003126	4.3 × 10^−6^	0.144		
17	−2038.779	2110.641	0.002246	0.002251	5.2 × 10^−6^	0.233	Particle Final Location	
18	−1211.095	1477.706	0.001013	0.001017	4.1 × 10^−6^	0.395		
19	−1089.377	820.429	0.000420	0.000421	1.2 × 10^−6^	0.202		
20	−1527.563	187.494	0.001102	0.001100	2.3 × 10^–6^	0.202		

The data are obtained for 70 µL droplet. *V*_*S*_ and *V*_*E*_ represent, velocities obtained from simulation and experiment, respectively.

## Results and Discussion

Heating of a sessile droplet on a hydrophobic surface are considered and the effect of column-like metallic pin located in the droplet fluid on the thermal fields is examined. Functionalized silica particles are deposited on the glass surface to obtain the hydrophobic surface with the droplet contact angle within the range of within 152° ± 2° to 156° ± 2°. PIV is utilized validating the velocity predictions inside the droplet.

### Droplet pinning on coated surfaces

The measurement of the contact angle of the coated surfaces is realized at several places on the sample. The measurements are conducted incorporating the procedure described in the previous study^25^. The measurement data reveal that the contact angle of the sample surface varies within 152° ± 2° to 156° ± 2° and the contact hysteresis is about 2°. Hence, the functionalized nanoparticles coated sample surfaces possess the superhydrophobic wetting state with low hysteresis. The droplet pinning on the coated surface requires sufficient adhesion-force in the longitudinal direction on the surface plane. The adhesion force in the plane of the surface is related to the force balance due to the surface tension and the gravitational forces along the coated sample surface. Since the droplet contact angle is large, the three phase contact length remains small on the nanoparticles coated surface. In addition, the small hysteresis angle has an adverse effect on the adhesion force. The adhesion force associated with the pinning of the droplet on the surface can be expressed as ${F}_{add}=\pi {D}_{w}\gamma fcos({\theta }_{R}-{\theta }_{A})$, where *D*_*w*_ represents the equivalent diameter of the contact line, *γ* corresponds to the surface tension, *f* resembles the roughness parameter, *θ*_*R*_ being the receding angle and *θ*_*A*_ is the advancing angle of the droplet. The adhesion force for 90 µL droplet is determined in the order of 2.1 × 10^−5^ N. Hence, in line with the force balance (${F}_{R}={F}_{add}-mgsin\delta$, where *F*_*R*_ is the inertia force, *m* is the droplet mass, and *δ* is the sample inclination angle), if the nanoparticles coated surface is inclined larger than 1.36°, then, the force due to gravity on the inclined surface (*mgsinδ*) remains greater than the adhesion force. Therefore, the inertial force of the droplet remains none-zero while causing the droplet movement on the inclined surface. Consequently, a consideration is made to locate the functionalized silica particles glass plate remaining horizontal during the experiments. Moreover, the flow field is created in the fluid due to heating from the plate and the pin. This results in development of the shear rate at the bottom of the droplet and along the vertical pin interface, which also adds to the dynamic instability of the droplet on the sample surface. The shear force (*F*_*τ*_) is estimated via ${F}_{\tau }={\int }_{0}^{{r}_{w}}\mu \frac{dV}{dn}2\pi r$, where *dV*/*dn* is the rate of fluid strain in the droplet normal to the wetted surface, which slightly changes across wetted diameter, and it is determined from the simulation data, *r*_*w*_ is the radius of the wetted area at the droplet bottom, i.e. ${r}_{w}=\frac{{D}_{w}}{2}$. Hence, the shear force is determined as about 2.3 × 10^−6^ N, which is less than the force of retention (1.8 × 10^−4^ N). The retention force is same order of adhesion force (*F*_*add*_) as formulated earlier. Hence, the droplet pins on the sample during heating. As the droplet is formed on the hydrophobic plate, it undergoes geometric bulging from circular to oval shapes, which is considered as the droplet puddling. However, the size of the droplet has an effect on the puddling of the droplet when formed on the hydrophobic surface. As the droplet characteristics diameter, which corresponds to the droplet diameter when it is in a circular shape, becomes less (such as in the order of few hundred micrometers for water droplet) than the capillary-length (${\kappa }^{-1}=\sqrt{\frac{\gamma }{\rho g}}$, where *κ*^−1^ represents the capillarity-length, *γ* corresponds to the surface tension, *ρ* is the density, and *g* being the gravitational acceleration), the droplet remains spherical on the sample with undergoing small bulging^31^. In this case, the droplet can roll/slide on the sample surface with considerably small surface inclinations. In the present study, the droplet characteristic diameters are in the order of couple of millimeters, which are larger than the capillary length of the droplet. Therefore, the droplet bugles and forms puddle on the hydrophobic surface. Moreover, once the metallic column pin, which has hydrophilic surface characteristics, is introduced into the droplet on the hydrophobic surface, the droplet pinning further improves on the surface. In this case, the wetting of the droplet fluid around the column pin contributes to the size of the three-phase contact line around the droplet wetting sites, i.e. droplet wetting takes place on the hydrophobic surface located at the droplet bottom and around the rim of the column pin introduced.

### Heating analysis in droplet interior

Since the velocity and temperature fields developed inside the droplet fluid show transient behavior, the simulations are carried out to assess the quasi-steady behavior of the temperature and velocity fields. Figure 5a shows temperature behavior along the horizontal line for various heating durations while Fig. 5b shows velocity variation along same horizontal line for various heating durations. Temperature and velocity distributions along the horizontal line become identical for 30 s and 40 s of the heating durations. Consequently, the thermal fields (flow/temperature) attain almost quasi-steady after 30 s of heating. Small heating duration is attributed to both the heating of droplet via column pin and the hydrophobic surface. Figure 6a depicts velocity in the free droplet (with the absence of the column pin) while Fig. 6b shows velocity in the droplet with the column pin for different droplet sizes. The optical images of the droplets are also shown in Fig. 6. For free-droplet, two counter rotating circulating cell-structures are developed within the droplet. Although surface evaporation under high temperature gradients or long durations alters the flow field^32^, the surface evaporation from the droplet becomes minimum due to low temperature gradient and short heating durations (40 s) in the present study. Moreover, evaporation of the surface is assessed from the droplet images of the high speed recording over 100 s. It is observed that the volume change of the droplet is about 0.15% over the period of 100 s. The Marangoni current developed, due to the surface tension gradient at the droplet fluid-air interface, and the buoyancy current formed via thermal expansion of the droplet fluid is responsible for the formation of the counter rotating circulation cells. Similar findings are also reported in the previous study; in which case, two counter rotating circulating cell-structures are formed inside the droplet subjected to a hot plate heating from droplet bottom^33^. The centers of circulation cells are formed in the upper region of the droplet, which are affected by both the Marangoni and the buoyancy forces. Due to heating, the temperature gradient attains larger values in bottom region than that corresponding to the droplet fluid interior. This in turn gives rise to thermal expansion of the droplet fluid in this region while resulting in the buoyancy current.

**Figure 7:**
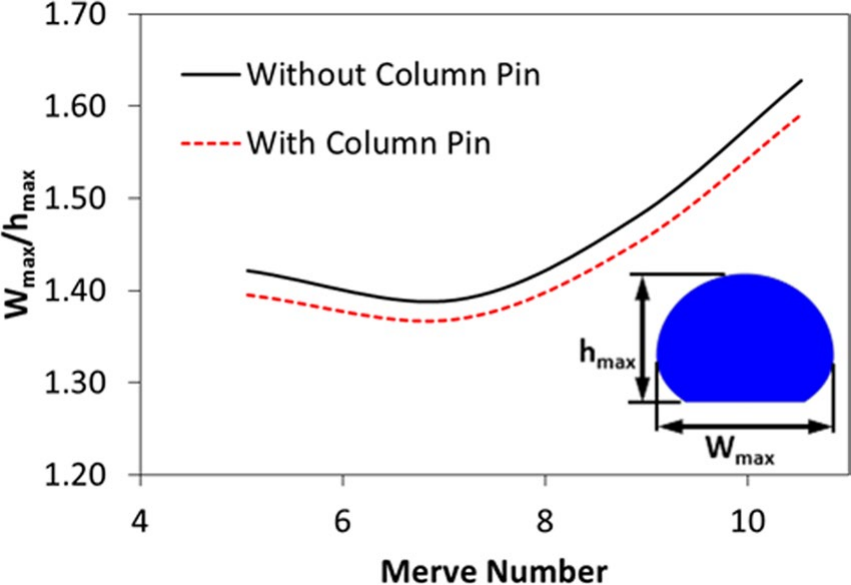
Geometric ratio of droplet with Merve number for droplet with pin and without pin configurations. W_max_ represents the maximum width of droplet and h_max_ is the maximum height of droplet.

The heated fluid contributes to lifting of the circulation cell centers towards the droplet upper region. This is apparent for the small droplets. As the droplet volume increases, the maximum velocity increases in the droplet, i.e. the droplet height and the area of the droplet free surface increase with increasing droplet volume. In addition, increasing droplet volume causes droplet bulging on the plate; hence, increase of the droplet height becomes comparable to the increase of the droplet maximum width. This can be observed from the ratio of the droplet maximum height with the droplet maximum width (Fig. 7). Consequently, the droplet width extends more than the droplet height with increasing volume. Nevertheless, the droplet surface area (fluid-air) enlarges with increasing droplet volume. Hence, the gradient of the surface tension temperature $(\frac{d\gamma }{dT})$ increases while enhancing the Marangoni current. In addition, the circulating cell-structure size increases with enlarging volume of the droplet. Therefore, increasing circulating cell-structure size and the surface tension gradient modify the center of the circulating cell-structure and the maximum velocity. In the case of the droplet with column pin (Fig. 6b), four counter rotating circulating cell-structures are formed inside the droplet. However, the size and orientation of the circulating structures change significantly from those corresponding to the free droplet case (Fig. 6a). Hence, the presence of the column pin results in breaking of the large circulation cells into four circulation cells. Since the heating occurs between the column pin and the fluid because of higher pin temperature, the buoyancy current generated in the close region of the pin-droplet fluid interface contributes to the breaking of the circulation cells into four cells inside the droplet fluid. As the droplet volume increases, the size of the circulation cells reduces in the region of the droplet bottom and the circulation cells almost disappears in this region for the droplet volume of 90 µL. Consequently, the presence of the column pin inside the droplet modifies significantly the flow field. As similar to Fig. 6a, enlarging droplet size gives rise to enhanced maximum velocity in the fluid. The presence of the column pin slightly alters the geometric ratio of the pin height over the maximum pin width (Fig. 7). 4 is Moreover, the droplet geometric changes modify slightly the droplet free surface area (at droplet fluid-air) and alter the surface tension temperature gradient $(\frac{d\gamma }{dT})$ while changing the Marangoni current developed inside the droplet fluid. Consequently, this also influences the breaking of the circulation cells inside the droplet.

**Figure 8:**
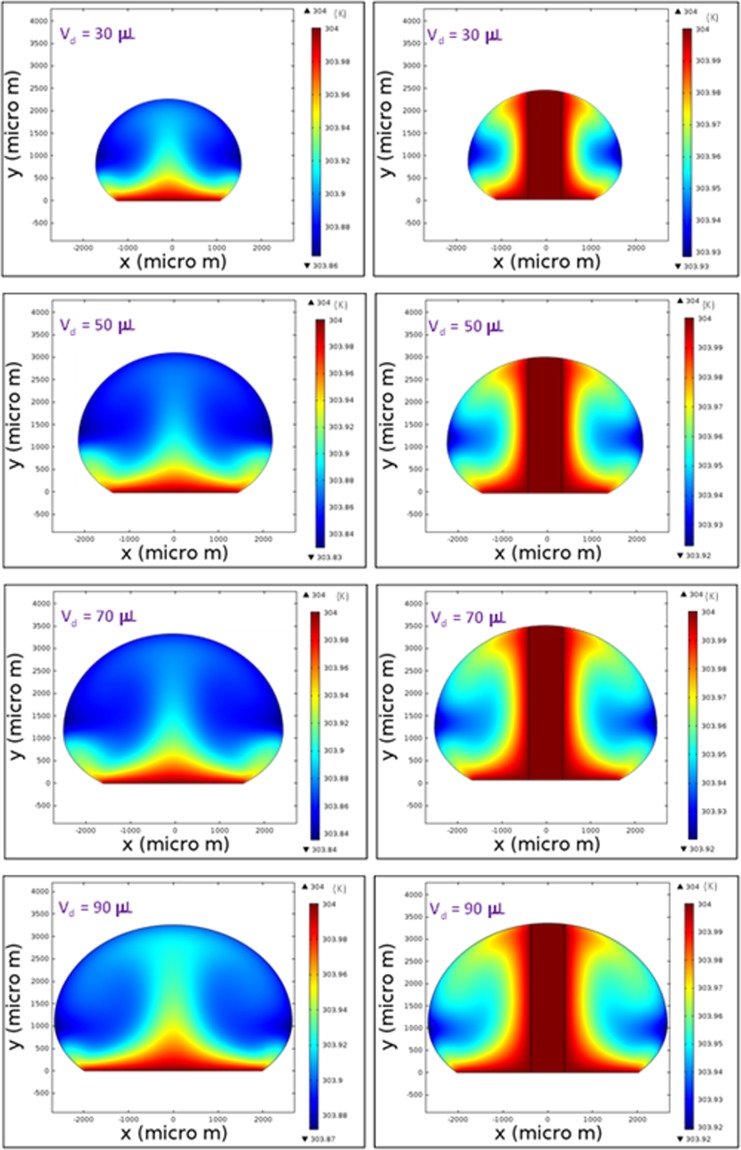
Temperature contours inside droplet with pin and without presence of pin configurations for various droplet volumes after 40 s of heating.

**Figure 9:**
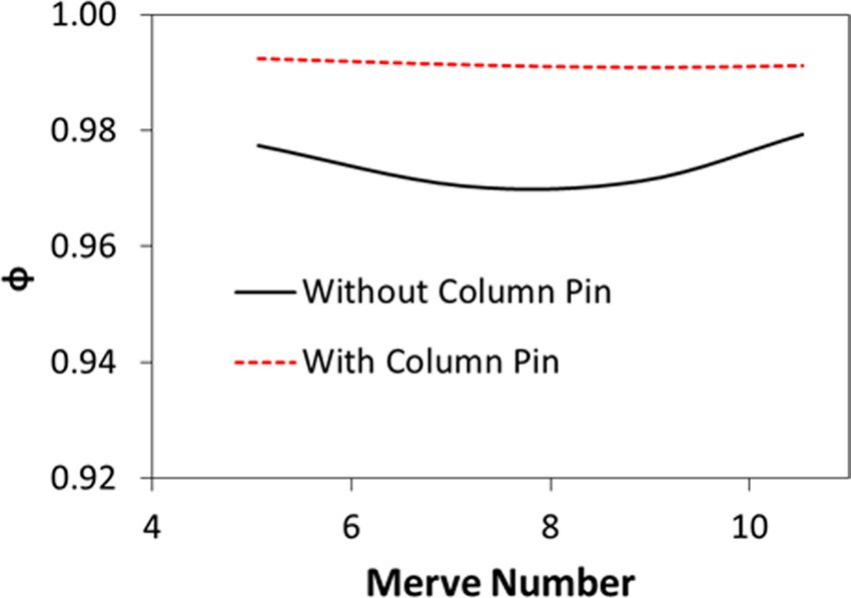
Temperature parameter ($\frac{{T}_{bulk}-{T}_{in}}{{T}_{max}-{T}_{in}}$, *T*_*bulk*_ is the fluid bulk temperature, *T*_*in*_ is the initial fluid temperature, *T*_*max*_ is the maximum fluid temperature) with Merve number for droplet with pin and without presence of pin configurations.

**Figure 10:**
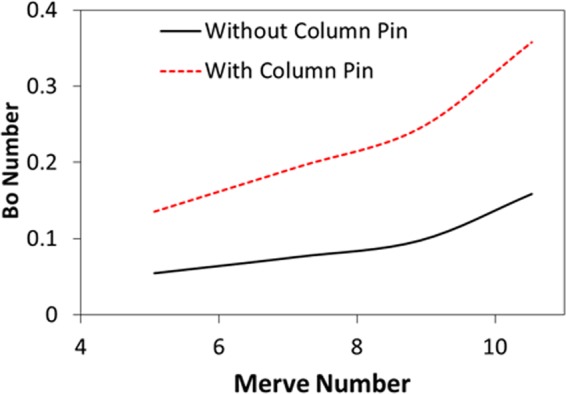
Bond number variation with Merve number for droplet with pin and without presence of pin configurations.

Figure 8 shows temperature variation in the droplet for the cases corresponding to a sessile droplet and the droplet with column pin for various droplet volumes. For the free droplet case, heating occurs from the droplet-plate interface towards the fluid interior. The heated fluid is carried by the buoyancy current to the region of the droplet-fluid-air interface. However, the circulation cell forms a close loop like flow field inside the droplet while keeping the droplet fluid temperature low in the region of the circulation cells. In addition, heat diffusion in the central region of the droplet enhances temperature increase in this region, which in turn extends the heated section in the droplet interior. Because of the counter rotating of the circulation cells, the shear layer developed in the circulation cell outer boundary lowers the flow velocity in this region. Consequently, the heated region extends in between the circulation cells outer boundary in the fluid, which becomes more apparent for large volumes. In the case of droplet with column pin. Heating occurs from the droplet bottom and from the column pin surface. This disturbs the flow field in the droplet and modifies the surface tension gradient and the thermal expansion of the droplet fluid, i.e. the Marangoni and the buoyancy currents modify the flow structure inside the droplet (Fig. 6b). Temperature remains high in the close region of the droplet bottom and the vicinity of the column pin because of the heat diffusion. Hence, the thermal expansion influences the buoyancy force in this region and alters the flow in the fluid (Fig. 6b). The heated fluid is transported via the buoyancy current towards the droplet fluid interior. In this case, temperature increases almost in all locations inside the droplet unlike the free droplet case. However, some small sections of the cold fluid are observed in the circulation cells region of the droplet; in which case, the closed-pack behavior of the circulation cell-structures inhibits the heated-fluid penetrating into this region. Temperature enhancement in the droplet fluid can also be seen from Fig. 9, in which the temperature parameter ($\phi =\frac{{T}_{bulk}-{T}_{in}}{{T}_{max}-{T}_{in}}$, where *T*_*bulk*_ is droplet fluid bulk temperature, *T*_*in*_ is droplet fluid initial temperature (300 K), *T*_*max*_ is maximum fluid temperature (304 K)) is shown. It should be noted that the fluid bulk temperature is computed from the mass average temperature. The temperature parameter attains larger values for the droplet with the column pin than the free-droplet. This shows that the fluid bulk temperature improves with including the column pin inside the droplet. Moreover, the Merve number represents the surface tension force over the gravitational force and it bases on the gravitational force corresponding to the initially formed droplet mass prior to heating, i.e. for a given droplet volume it remains constant. In addition, due to the density variations in the droplet fluid and surface evaporation, the Bond number takes different values for a given droplet volumes during the heating period. Hence, it is more appropriate to use the initial droplet weight calculating the force ratio due to surface tension over the gravitational force. In addition, temperature parameter varies with the Merve number for the free-droplet case while it remains almost constant for the droplet with column pin case. Consequently, the size effect of the droplet on the fluid bulk temperature is more significant for the free droplet case than that of the droplet with column pin case. Figure 10 depicts the Bond number with the Merve number. The Bond number rises with increasing Merve number. Since the Bond number represents the qualitative ratio of buoyancy current over the Marangoni current, increasing the droplet volume improves the buoyancy current in the fluid. This situation is more pronounced for the large Merve numbers (large size droplets). However, the Bond number attains larger values for the column pin inside the droplet fluid than that of the free-droplet. This behavior is attributed to the buoyancy current generated in the near region of the high temperature column pin. Nevertheless, the Bond number remains less than unity for free the droplet and the droplet with column pin configurations. This demonstrates that Marangoni current domination occurs in the droplet fluid, despite the variation of the buoyancy current with the droplet volume. It should be noted that for binary fluids with low evaporating temperature fluid such as ethanol and n-butanol, density variation in the droplet becomes critical generating the buoyancy current. In addition, surface evaporation significantly influences the surface tension gradient and Marangoni influence becomes less on the droplet internal flow, particularly for non-uniform flow, i.e., for nonuniform evaporation across the surface, the convection dominates over the diffusion^3^.

**Figure 11:**
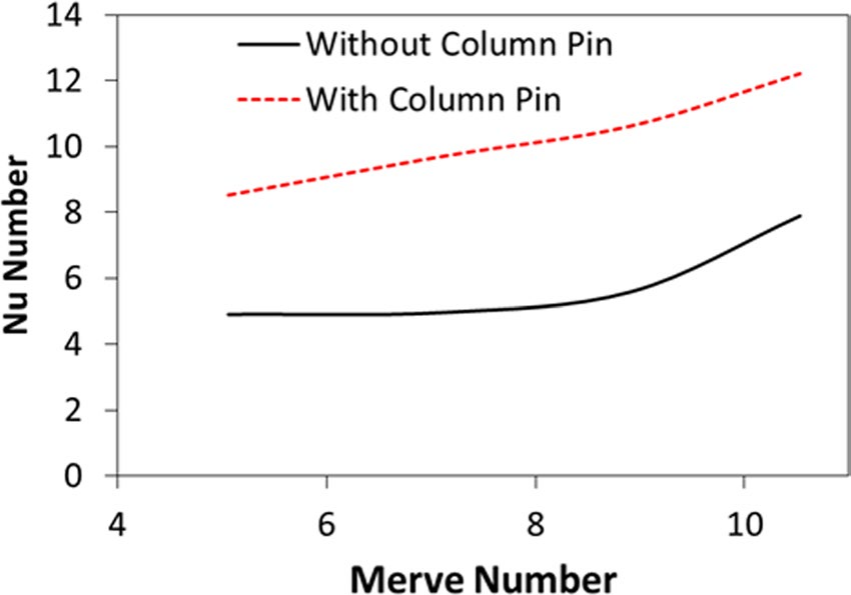
Nusselt number variation with Merve number for droplet with pin and without presence of pin configurations.

Figure 11 depicts the Nusselt number with the Merve number for the free droplet and the droplet with column pin configurations. The Nusselt number becomes larger for the column pin configuration than that of the free droplet. This behavior is attributed to the enhanced heat diffusion from the column pin to the droplet fluid and the heat convection due to the Marangoni and the buoyancy currents. In addition, increasing Merve number (for large droplet size) enhances the maximum value of the convection current in the droplet (Fig. 6b) while improving the convection heat transfer in the droplet fluid; hence, the Nusselt number increases with increasing Merve number. Nevertheless, the Nusselt number enhancement with the increase of the Merve number remains almost in a linear manner for the column pin arrangement. Therefore, the droplet size increase gives rise to the Nusselt number increase.

## Conclusions

Heat transfer and fluid flow in a droplet placed on the hydrophobic plate are studied and the influence of the vertically located (column) pin on the thermal fields inside the fluid is examined. In the first case, the free droplet is heated from the hydrophobic plate at a constant temperature while in the second case, heating is modified via introducing a column pin inside the droplet as the additional heating source. This arrangement provides insight information on the droplet heating with extended surface. The study is extended to include the effect of the droplet size on the thermal behavior inside the droplet fluid. An experiment is realized using the particle image velocimetry (PIV) validating the velocity field simulated inside the droplet. It is observed that the velocity predictions agree well with the experimental findings obtained from the PIV measurements. Heating from the plate results in development of the Marangoni and the buoyancy currents inside the free droplet, which in turn generate two counter-circulating structures in the droplet fluid. The size and the center of the circulating cells differ with the droplet volume. The heat diffusion increases temperature in the near region of the droplet bottom and the buoyancy current carries the heated fluid towards the droplet sides. However, the circulation cells behave-like close-packed structures while reducing the fluid with high temperature moving to the circulation cells region in the droplet fluid. Introducing the column pin inside the droplet fluid modifies the circulation cell size and increases the number of the circulation cells to four. This is related to the modification of the buoyancy and Marangoni currents via heating from the column pin to the fluid. Increasing the droplet volume improves the maximum velocity in the fluid, which becomes apparent for the column pin arrangement. The column pin enhances the Nusselt number significantly and the Nusselt Number increase becomes considerable as the droplet volume increases. The Bond number also enhances with the droplet size and the increase becomes considerable after including the vertical column pin inside the droplet, which is associated with increased buoyancy current in the near zone of the column pin. However, the Bond number values are less than unity for the free droplet and the droplet with column pin configurations. This demonstrates that the effect of the Marangoni current on the flow and temperature fields is significant regardless of heat transfer enhancement via introducing the column pin inside the droplet. The present study gives insight into the droplet heat transfer and provides information on the enhancement of the droplet heating with introducing the column pin inside the droplet.

## References

[1] Tam D, von ARNIM V, McKinley G, Hosoi A (2009). Marangoni convection in droplets on superhydrophobic surfaces. Journal of Fluid Mechanics.

[2] Lu G, Duan Y-Y, Wang X-D, Lee D-J (2011). Internal flow in evaporating droplet on heated solid surface. International journal of heat and mass transfer.

[3] Edwards A (2018). Density-driven flows in evaporating binary liquid droplets. Physical review letters.

[4] Yang Z, Ma X-C, Duan Y-Y, Chen Y (2013). Internal flow and heat transfer of a condensing water droplet in steam flow. Chemical Engineering Science.

[5] Wang K, Ye J, Bai B (2017). Entrained droplets in two-phase churn flow. Chemical Engineering Science.

[6] Misyura S (2017). Contact angle and droplet heat transfer during evaporation on structured and smooth surfaces of heated wall. Applied Surface Science.

[7] Teodori E, Pontes P, Moita A, Moreira A (2018). Thermographic analysis of interfacial heat transfer mechanisms on droplet/wall interactions with high temporal and spatial resolution. Experimental Thermal and Fluid Science.

[8] Legros JC, Piskunov MV (2018). Evaporation of water droplets with metallic inclusions. International journal of multiphase flow.

[9] Al-Sharafi A (2017). Internal flow and heat transfer in a droplet located on a superhydrophobic surface. International Journal of Thermal Sciences.

[10] Al-Sharafi A, Yilbas BS, Ali H (2018). Heat and flow analysis of a water droplet on hydrophobic and hydrophilic phase change material. International Journal of Heat and Mass Transfer.

[11] Gibbons M, Di Marco P, Robinson AJ (2018). Local heat transfer to an evaporating superhydrophobic droplet. International Journal of Heat and Mass Transfer.

[12] Al-Sharafi A, Yilbas BS, Ali H (2017). Droplet heat transfer on micro-post arrays: Effect of droplet size on droplet thermal characteristics. International Journal of Heat and Fluid Flow.

[13] Phadnis A, Rykaczewski K (2017). The effect of Marangoni convection on heat transfer during dropwise condensation on hydrophobic and omniphobic surfaces. International Journal of Heat and Mass Transfer.

[14] Al-Sharafi A, Yilbas BS, Sahin AZ, Ali H, Al-Qahtani H (2016). Heat transfer characteristics and internal fluidity of a sessile droplet on hydrophilic and hydrophobic surfaces. Applied Thermal Engineering.

[15] Wu Y, Zhang X, Zhang X (2016). Simplified analysis of heat and mass transfer model in droplet evaporation process. Applied Thermal Engineering.

[16] Maneshian B, Javadi K, Rahni MT, Miller R (2016). Droplet dynamics in rotating flows. Advances in colloid and interface science.

[17] Wen R (2017). Hydrophobic copper nanowires for enhancing condensation heat transfer. Nano Energy.

[18] Sim J, Im HG, Chung SH (2015). A computational study of droplet evaporation with fuel vapor jet ejection induced by localized heat sources. Physics of Fluids.

[19] Krahl, R., Adamov, M. & Avilés, M. L. A model for two phase flow with evaporation. (2004).

[20] Semenov S, Starov VM, Rubio RG, Velarde MG (2012). Computer simulations of evaporation of pinned sessile droplets: influence of kinetic effects. Langmuir.

[21] George OA (2017). Detailed numerical analysis of evaporation of a micrometer water droplet suspended on a glass filament. Chemical Engineering Science.

[22] http://www.comsol.com/comsol-multiphysics. (2019).

[23] Mackenzie J, Mekwi W (2011). An unconditionally stable second-order accurate ALE–FEM scheme for two-dimensional convection–diffusion problems. IMA Journal of Numerical Analysis.

[24] Yong WYD, Zhang Z, Cristobal G, Chin WS (2014). One-pot synthesis of surface functionalized spherical silica particles. Colloids and Surfaces A: Physicochemical and Engineering Aspects.

[25] Heib F, Schmitt M (2016). Statistical contact angle analyses with the high-precision drop shape analysis (HPDSA) approach: Basic principles and applications. Coatings.

[26] Lin J, Chen H, Ji Y, Zhang Y (2012). Functionally modified monodisperse core–shell silica nanoparticles: Silane coupling agent as capping and size tuning agent. Colloids and Surfaces A: Physicochemical and Engineering Aspects.

[27] Smith JD (2013). Droplet mobility on lubricant-impregnated surfaces. Soft Matter.

[28] Al-Sharafi A, Sahin AZ, Yilbas BS, Shuja S (2016). Marangoni convection flow and heat transfer characteristics of water–CNT nanofluid droplets. Numerical Heat Transfer, Part A: Applications.

[29] Al-Sharafi A, Yilbas BS, Ali H (2017). Heat transfer and fluid flow characteristics in a sessile droplet on oil-impregnated surface under thermal disturbance. Journal of Heat Transfer.

[30] De Santo M, Liguori C, Pietrosanto A (2000). Uncertainty characterization in image-based measurements: a preliminary discussion. IEEE transactions on instrumentation and measurement.

[31] Aussillous P, Quéré D (2004). Shapes of rolling liquid drops. Journal of Fluid Mechanics.

[32] Yu YS, Xia XL, Zheng X, Huang X, Zhou JZ (2017). Quasi-static motion of microparticles at the depinning contact line of an evaporating droplet on PDMS surface. Science China Physics, Mechanics & Astronomy.

[33] Wang Z, Zhao YP (2012). *In situ* observation of thermal Marangoni convection on the surface of a sessile droplet by infrared thermal imaging. Journal of adhesion science and technology.

